# Inhibition of PAI-1 shifts cardiomyocyte fate from senescence toward apoptosis and mitigates doxorubicin-induced cardiotoxicity

**DOI:** 10.70401/geromedicine.2026.0018

**Published:** 2026-03-17

**Authors:** Yuka Shiheido-Watanabe, Eun-Ah Sung, Andreas Ivessa, Peiyong Zhai, Takuma Takada, Soichiro Ikeda, Masato Matsushita, Daniela Zablocki, Junichi Sadoshima

**Affiliations:** Department of Cell Biology and Molecular Medicine, Cardiovascular Research Institute, Jersey Medical School, 185 South Orange Ave, Newark, NJ 07103, USA.

**Keywords:** Doxorubicin, PAI-1, BioID, TM5275, senescence

## Abstract

**Aims:**

Doxorubicin (Dox) is an effective chemotherapeutic agent, but its clinical use is limited by cardiotoxicity. Cellular senescence contributes to Dox-induced cardiac dysfunction; however, the underlying molecular mechanism mediating the effect of senescence remains poorly understood. This study aimed to identify senescence-associated factors secreted from cardiomyocytes in Dox-treated hearts and define their functional significance in Dox-induced cardiotoxicity.

**Methods:**

Mice with cardiomyocyte-specific expression of the endoplasmic reticulum BioID secretome profiling system were used to identify Dox-induced secreted factors. Functional analyses were performed in neonatal rat ventricular myocytes (NRVMs). The effects of plasminogen activator inhibitor-1 (PAI-1) inhibition were evaluated in Dox-treated mice by assessing senescence markers, apoptotic responses, and cardiac structure and function. *p21*^*High*^-*tdTomato* reporter mice were used to examine the fate of senescent cardiomyocytes *in vivo*.

**Results:**

PAI-1 was identified as a major component of the senescence-associated secretory phenotype and was robustly upregulated in Dox-treated cardiomyocytes. In NRVMs, PAI-1 promoted senescence and maintained the senescent phenotype, in part by conferring resistance to apoptosis. Pharmacological inhibition of PAI-1 reduced senescence markers, enhanced apoptotic responses, and preserved cardiac structure and function in Dox-treated mice. Fate mapping analyses with *p21*^*High*^-*tdTomato* mice revealed that PAI-1 inhibition decreased the number of *p21*^*High*^ senescent cardiomyocytes in Dox-treated hearts. Notably, PAI-1 inhibition did not attenuate Dox cytotoxicity in EO771 murine breast cancer cells.

**Conclusion:**

PAI-1 is a key mediator of Dox-induced cardiac dysfunction. PAI-1 inhibition shifts the fate of cardiomyocytes from senescence toward apoptosis and preserves cardiac structure and function without compromising the antitumor function of Dox, highlighting PAI-1 as a potential therapeutic target for chemotherapy-associated cardiotoxicity.

## 1. Introduction

Doxorubicin (Dox), an anthracycline chemotherapeutic agent widely used in treating hematological and solid tumors, remains a cornerstone of modern cancer therapy. However, its clinical application is significantly limited by its cumulative and dose-dependent cardiotoxicity^[1]^. Cardiac dysfunction may manifest during treatment or emerge years after therapy, contributing substantially to long-term morbidity and non-cancer mortality in cancer survivors. Despite extensive studies implicating oxidative stress, mitochondrial dysfunction, and DNA damage in Dox-induced cardiomyopathy, effective strategies to prevent or reverse this complication remain elusive^[2]^.

Emerging evidence suggests that cellular senescence plays a pivotal role in driving Dox-induced cardiotoxicity^[3,4]^. Senescence in cardiomyocytes, characterized by telomere damage and the secretion of pro-inflammatory and pro-fibrotic factors, collectively known as the senescence-associated secretory phenotype (SASP), has been shown to disrupt myocardial structure and function^[5,6]^. However, the precise molecular mediators that orchestrate the establishment, maintenance, and paracrine effects of cardiomyocyte senescence under Dox stress remain incompletely understood. Autocrine and paracrine factors produced through the SASP are both cell-type and stimulus-specific. To our knowledge, the SASP factors specifically produced by and secreted from cardiomyocytes are poorly understood^[7]^. To address this question, we used cardiac-specific endoplasmic reticulum (ER)-BioID (*cER-BioID*) mice, in which a biotin conjugation enzyme is expressed on the endoplasmic reticulum membrane^[8]^. In these mice, proteins destined to either the plasma membrane or secretion are biotinylated in the presence of biotin. The expression of the ER-BioID system in a cardiomyocyte-specific manner in *cER-BioID* mice makes it possible to identify the factors secreted from cardiomyocytes in response to Dox. One factor we identified in the serum of Dox-treated mice was plasminogen activator inhibitor-1 (PAI-1).

PAI-1, a member of the serine protease inhibitor (serpin) superfamily, has been implicated as a SASP component in non-cardiac tissue, where it contributes to senescence and tissue remodeling^[9–11]^. Whether PAI-1 plays a similar role in the heart, particularly in response to genotoxic chemotherapy, remains unknown. Furthermore, the origin of PAI-1 in the serum and whether it is produced in senescent cells are unknown.

In this study, we aimed to define the role of PAI-1 in Dox-induced cardiac senescence and dysfunction. By employing the *cER-BioID* system in both *in vivo* and *in vitro* models of Dox-induced cardiomyopathy and pharmacological PAI-1 inhibition, we investigated whether PAI-1 serves as a key mediator of cellular senescence in Dox-induced cardiomyopathy. We further explored whether targeting PAI-1 alters the balance between senescence and apoptosis in cardiomyocytes and improves cardiac function. These findings may offer a novel therapeutic strategy to prevent or reverse Dox-induced cardiotoxicity.

## 2. Materials and Methods

### 2.1 Animals

C57BL/6J mice were purchased from The Jackson Laboratory (Bar Harbor, ME). *cER-BioID* mice were generated by crossing *stop*- *flox-ER-BioID*^*HA*^ knock-in mice (Jax 036263)^[8]^ with *Myh6-Cre* mice. *p21*^*High*^-*ER-BioID* mice were generated by crossing *stop*-*flox-ER-BioID*^*HA*^ knock-in mice with *p21*^*High*^-*Cre-ERT2* mice^[12]^. The *p21*^*High*^-*CreERT2* mice were generated by the Genome Editing Shared Resource at Rutgers, The State University of New Jersey, using previously published designs^[12]^. *p21*^*High*^-*tdTomato* mice were generated by crossing *Rosa-CAG-LSL-tdTomato* mice (Jackson Laboratory, #007914) with *p21*^*High*^-*Cre-ERT2* mice. Mice were housed at 22 °C with an alternating 12:12 hours light–dark cycle. Cages and food were changed once per week. Animals had free access to food and water *ad libitum*. All experiments involving genetically modified mice were performed using age-matched male and female mice. All experiments involving wild-type mice were performed using age-matched male mice. All animal protocols were approved by the Institutional Animal Care and Use Committee of the New Jersey Medical School, Rutgers, The State University of New Jersey. The investigators were blinded to the genotype groups during the experiments.

#### 2.1.1 Dox administration to mice

Phosphate-buffered saline (PBS)-dissolved Dox (Sigma) was administered to mice via intraperitoneal injection at a dose of 5 mg/kg once a week for 2 consecutive weeks^[13,14]^. Mice injected with PBS were used as controls.

#### 2.1.2 Biotin administration to mice

For *in vivo* secretome labeling, mice were administered a biotin solution via intraperitoneal and subcutaneous injections. Each mouse received 500 μL of a 2 mg/mL biotin solution (Sigma-Aldrich) once a day for five consecutive days^[8]^. In parallel, the mice were fed a biotin-supplemented diet during the same period. Serum samples were collected by terminal bleeding, and 50 μL of serum from each mouse was incubated with 20 μL of streptavidin-conjugated beads at 4 °C for 2 hours. After washing, the biotinylated proteins were eluted in 2× sodium dodecyl sulfate (SDS) loading buffer and analyzed by mass spectrometry.

#### 2.1.3 ABT-263 (Navitoclax) administration to mice

ABT-263 (Navitoclax, Selleckchem), dissolved in ethanol/polyethylene glycol 400/Phosal 50 PG at a ratio of 10:30:60, was administered to mice via oral gavage at a dose of 50 mg/kg body weight per day for five consecutive days^[15]^. Control mice received the vehicle solution only.

#### 2.1.4 TM5275 administration to mice

TM5275 (MedChemExpress LLC, NJ), suspended in 0.5% carboxymethyl cellulose sodium salt solution, was administered by oral gavage at a dose of 10 mg/kg body weight per day for five days per cycle^[16]^. Control mice received the vehicle solution only.

#### 2.1.5 Tamoxifen administration to mice

*p21*^*High*^-*ER-BioID* and *cp21-tdTomato* mice were fed a tamoxifen-containing diet for two weeks, after which they were switched to a regular diet.

### 2.2 Single cell RNA sequencing

Dox (5 mg/kg/week) was administered intraperitoneally twice. Control mice were injected with PBS. One week after the final injection, adult mouse heart cells were isolated from both PBS-treated (*n* = 3) and Dox-treated (*n* = 3) mice according to previously published protocols^[17]^. Freshly isolated cardiomyocytes and non-myocytes were pooled into PBS and Dox treatment groups and immediately fixed using Evercode Fixation v2 kits (Parse Biosciences), following the manufacturer’s instructions. Cells were processed to target a total of 10,000 cells across two sublibraries using the Evercode WT Mini v2 kit (Parse Biosciences). Barcoding and library generation were performed according to the manufacturer’s protocols. For data processing, sequencing reads were aligned using the ParseBioscience processing pipeline with default settings set to the GRCm38 mouse genome. Downstream analysis was conducted using the Seurat package in R, following standard procedures. Briefly, aligned count matrices were imported into an AnnData object and subjected to quality control filtering to exclude low-quality cells based on thresholds for gene counts, UMI counts, and mitochondrial gene content. The data were normalized to 10,000 counts per cell and log transformed. Highly variable genes were selected using sc.pp.highly_variable_genes, excluding mitochondrial and ribosomal genes. The data were scaled after regressing out total counts. Principal component analysis was performed on the variable genes. Cell-cell relationships were captured using a neighborhood graph (sc.pp.neighbors), and clusters were identified using the Leiden algorithm (sc.tl.leiden). UMAP was used for two-dimensional embedding. Differential gene expression analysis between clusters was performed using the Wilcoxon rank-sum test implemented in sc.tl.rank_genes_groups. All visualizations were generated using Scanpy’s built-in plotting functions and the matplotlib.

### 2.3 Primary culture of neonatal rat ventricular cardiomyocytes (NRVMs)

Primary cultures of ventricular cardiomyocytes were prepared from 1-day-old Charles River Laboratories rats and maintained in culture as described previously^[18]^. A cardiomyocyte-rich fraction was obtained by centrifugation, through a discontinuous Percoll gradient as described.

### 2.4 SA-β-galactosidase (SA-β-gal) assay

NRVMs were seeded in 12-well plates. After 24 hours, the medium was replaced with fresh medium and the cells were treated with Dox (100 nM) and/or TM5275 (10 μM). After 72 hours, β-galactosidase activity was assessed using the Senescence β-Galactosidase Staining Kit (Cell Signaling Technology) according to the manufacturer’s instructions. Briefly, the cells were washed once with 1× PBS and fixed with fixative solution at room temperature for 15 minutes. The cells were then washed with 1× PBS and stained with β-galactosidase staining solution at 37 °C in a dry incubator (without CO_2_) overnight. Cells were observed under a light microscope for the presence of blue precipitate. The percentage of positive cells was calculated by counting the blue-stained cells divided by the total number of cells for each sample.

### 2.5 Cell viability assay

EO771 murine breast cancer cells were seeded in 96-well plates at a density of 2 × 10^3^ cells per well. Cells were treated with Dox (100 nM) and/or TM5275 (25 μM). After 48 hours of treatment, cell viability was assessed using CellTiter-Blue^™^ (Promega) according to the manufacturer’s instructions. Fluorescence was measured using a synergy H1 plate reader (BioTek) with excitation at 560 nm and emission at 590 nm.

### 2.6 Echocardiography

Echocardiography was performed using a high-resolution Micro-Ultrasound system (Vevo 3100, FUJIFILM Visual-Sonic Inc., Toronto, Canada). Two-dimensional guided M-mode measurements of left ventricular internal diameter were obtained from at least three beats and then averaged. Left ventricular end-diastolic dimension (LVEDD) was measured at the time of the apparent maximal left ventricular diastolic dimension, and left ventricular end-systolic dimension (LVESD) was measured at the time of the most anterior systolic excursion of the posterior wall. Left ventricular ejection fraction (LVEF) was calculated using the following formula: LVEF (%) = 100 × (LVEDD^3^-LVESD^3^)/LVEDD^3[18]^.

### 2.7 Immunoblotting analysis

Cardiac tissue homogenates were prepared from the left ventricle apex. Cell lysates were prepared from primary cultures of rat ventricular myocytes. Both the homogenates and the cell lysates were prepared in a radioimmunoprecipitation assay (RIPA) buffer containing 150 mM NaCl, 1% Triton-X 100, 0.5% sodium deoxycholate, 0.1% sodium dodecyl sulphate, and 50 mM Tris (pH 8.0), and supplemented with a protease inhibitor cocktail (Sigma), 5 mM NaF, and 1 mM sodium orthovanadate. The following antibodies were used: phospho-H2AX (Cell Signaling Technology (CST), #9718), GAPDH (CST, #2118), PAI-1 (CST, #2753), cleaved caspase-3 (CST, #9664), p21 (Santa Cruz Biotechnology, #SC-1661), p53 (Bioss, #8687R), p16 (Invitrogen, #MA5–17142), ATM (CST, #2837), pATM (CST, #4526) and secondary antibodies (anti-rabbit or anti-mouse horseradish peroxidase-conjugated antibodies, CST, #7074 and #7076).

### 2.8 Pull-down assays

Streptavidin–agarose (Thermo Fisher Scientific) was added to 50 μL of serum and gently rotated at 4 °C for 2 hours. The streptavidin–agarose was then washed three times with PBS and eluted with 2× sample buffer. The eluates were subjected to SDS-PAGE and either stained with Coomassie brilliant blue or immunoblotted.

### 2.9 Mass spectrometry analyses

Streptavidin–agarose (Thermo Fisher Scientific) was added to 50 μL of serum and gently rotated at 4 °C for 2 hours. The streptavidin–agarose was then washed three times with PBS and eluted with 2× sample buffer, and the eluates were subjected to SDS-PAGE and stained with Coomassie brilliant blue. Each gel lane was excised and in-gel trypsin digestion was performed. The resulting peptides were analyzed by LC-MS/MS on a Q Executive MS instrument (Thermo Scientific, Canoga Park, CA).

### 2.10 TUNEL assays

NRVMs were fixed in PBS containing 4% paraformaldehyde. Staining was performed using the In situ Cell Death Detection kit (Roche) as described^[19]^. Nuclear density was determined by manual counting of DAPI-stained nuclei in six fields for each animal using the 40× objective, and the number of TUNEL-positive nuclei was counted by examining the entire section using the same power objective. Limiting the counting of total nuclei and TUNEL-positive nuclei to areas with a true cross-section of myocytes made it possible to selectively count only those nuclei that clearly were within myocytes^[19]^.

### 2.11 SDS-PAGE and Western blotting

Heart homogenates and cardiomyocyte lysates for SDS-PAGE and immunoblotting analyses were prepared in RIPA lysis buffer containing 50 mM Tris (pH 7.5), 150 mM NaCl, 0.1% SDS, 1% Triton X-100, 1% sodium deoxycholate, 1 mM EDTA, 1 mM sodium orthovanadate, 1 mM sodium fluoride, and 1× Halt Protease inhibitor cocktail (Thermo Fisher Scientific). After determining the protein concentrations by BCA assay, equal amounts of protein were loaded on an SDS-PAGE gel with 4× Laemmli sample buffer (200 mM Tris-HCl (pH 6.8), 40% glycerol, 8% SDS, 0.4% bromophenol blue, 10% β-mercaptoethanol). For separation under non-reducing conditions, β-mercaptoethanol was not added to the loading buffer. Proteins were then transferred to a PVDF membrane and immunoblotting was carried out using relevant antibodies. Densitometric analyses of the blots were carried out using the public domain ImageJ program (NIH).

### 2.12 Gene silencing via small interfering RNAs (siRNA) transfection

siRNAs were transfected into cells using Lipofectamine RNAiMAX (Thermo Fisher Scientific). Pre-designed Silencer Select siRNA targeting rat Serpine1 (s128154) and non-targeting control siRNA (siControl, #4390843) were purchased from Thermo Fisher Scientific. Lipofectamine RNAiMAX (Thermo Fisher Scientific) was first diluted in Opti-MEM medium (Thermo Fisher Scientific) and subsequently mixed with each siRNA. The mixture was incubated for 15 minutes at room temperature to allow complex formation. The resulting Lipofectamine–siRNA complexes were added to cardiomyocyte cultures, followed by gentle agitation of the mixture. After 24 hours, the transfection medium was replaced with serum-free DMEM/F-12 containing penicillin/streptomycin. Cells were subsequently cultured under standard conditions for downstream analyses.

### 2.13 Immunofluorescence analyses

Mouse hearts were harvested, fixed with 10% formalin and embedded in wax, and cross-sections (5 μm thick) were prepared. Neonatal cardiomyocytes were cultured on coverslips and fixed with 4% paraformaldehyde. An overnight incubation with specific antibodies against phospho-H2AX, IL-6 (Thermo Fisher Scientific, #P620), RFP (Rockland, #600-401-379), and cTNT (Invitrogen, #MA5–12960) was followed by a 2-hour incubation with secondary antibody conjugated with Alexa Fluor 488, 568, or 647 dye (Life Technologies). Samples were washed and mounted with a reagent containing DAPI (VECTASHIELD; Vector Laboratories). The analyses were performed by fluorescence microscopy in a blinded manner. For analysis of γH2AX-positive cardiomyocytes, the total number of γH2AX-positive nuclei in cardiomyocytes in the whole section was counted. The total number of cardiomyocytes in the whole section was estimated as the product of the average number of cardiomyocytes per image and the total number of images per section. The percentage of γH2AX-positive cardiomyocytes was calculated by the equation: (the number of γH2AX-positive cardiomyocytes/total number of cardiomyocytes in the section) × 100^[14]^.

### 2.14 Cell death detection

NRVMs were seeded in 4-well chamber slides and cultured overnight. The medium was then replaced with fresh medium and the cells were treated with Dox (100 nM) and/or TM5275 (25 μM) for 3 hours. Apoptosis and necrosis were detected using the Cell Meter^™^ Apoptotic and Necrotic Detection kit (AAT Bioquest, Inc., Sunnyvale, CA) according to the manufacturer’s protocol. The cells were observed and analyzed in a blinded manner using fluorescent microscopy.

### 2.15 Statistical analyses

All data are expressed as the mean ± SEM. All statistical analyses were performed using an unpaired Student’s *t*-test or one-way ANOVA followed by a *post hoc* Bonferroni-Dunn’s comparison test for multiple group comparisons, or two-way ANOVA followed by Sidak’s multiple comparison test. A value of *P* < 0.05 was considered significant.

## 3. Results

### 3.1. Dox treatment enhances cardiomyocyte-derived PAI-1 secretion in *cER-BioID* mice

To characterize the cardiomyocyte-specific secretome during the acute phase of Dox-induced cardiomyopathy, we generated *cER-BioID* mice by crossing *stop-flox-ER-BioID*^*HA*^ mice with *Myh6-Cre* mice (Figure S1A). The ER-BioID protein localizes to the ER, enabling the biotinylation of proteins destined for classical secretion^[8]^. This system permits selective temporal labeling of secreted proteins derived specifically from cardiomyocytes (Figure S1B,C). *cER-BioID* mice exhibited a normal cardiac phenotype at baseline (Figure S2A).

Our previous study demonstrated that repeated administration of Dox (5 mg/kg/week, four times) induces cardiac dysfunction and cardiomyocyte senescence in mice^[14]^. To capture the cardiomyocyte-specific secretome associated with Dox-induced cardiac dysfunction, *cER-BioID* mice were treated with Dox (5 mg/kg) or PBS on Day 0 and on Day 7, the point at which LVEF typically begins to decline^[14]^. We confirmed that Dox treatment induced cardiac dysfunction in *cER-BioID* mice similar to that in wild type mice as reported previously^[14]^ (Figure S2B). Biotin supplementation was administered via intraperitoneal (i.p.) injections and biotin-enriched chow for five consecutive days beginning after the second Dox dose (on Day 8, 500 μL of 2 mg/mL biotin) (Figure S1D). Serum samples were then collected on Day 14, and biotinylated proteins were isolated using streptavidin-conjugated beads and analyzed via mass spectrometry (MS). Proteins upregulated in the Dox + biotin group relative to both the PBS + biotin and the PBS without biotin groups were identified as candidate cardiomyocyte-specific Dox-induced secreted factors (Figure S3A,B). Of these proteins, two molecules in the serine protease inhibitor superfamily (serpins), namely PAI-1 (*Serpine1*) and alpha-1-antitrypsin 1–5 (*Serpina1e*), were the most elevated (Figure S3A,B). The Search Tool for the Retrieval of Interacting Genes/Proteins network analysis highlighted that serpins have functional interactions with other proteins identified in the screen, including Murinoglobulin-1, Haptoglobin and Leucine-rich HEV glycoprotein (Figure S3C). Serpins have diverse functions and are involved in blood coagulation, fibrinolysis, programmed cell death, development and inflammation^[9]^. PAI-1 mediates Dox-induced senescence in alveolar type II cells and cultured cardiomyocytes^[10,11]^. However, the involvement of PAI-1 in Dox-induced cardiomyopathy *in vivo*, particularly its function as a SASP factor, the cell type that produces PAI-1, and its functional significance are unknown. Given the known ability of PAI-1 to induce tissue remodeling^[20]^, we focused on PAI-1 in this study and hypothesized that it is secreted from senescent cardiomyocytes in response to Dox treatment.

**Figure 1.**
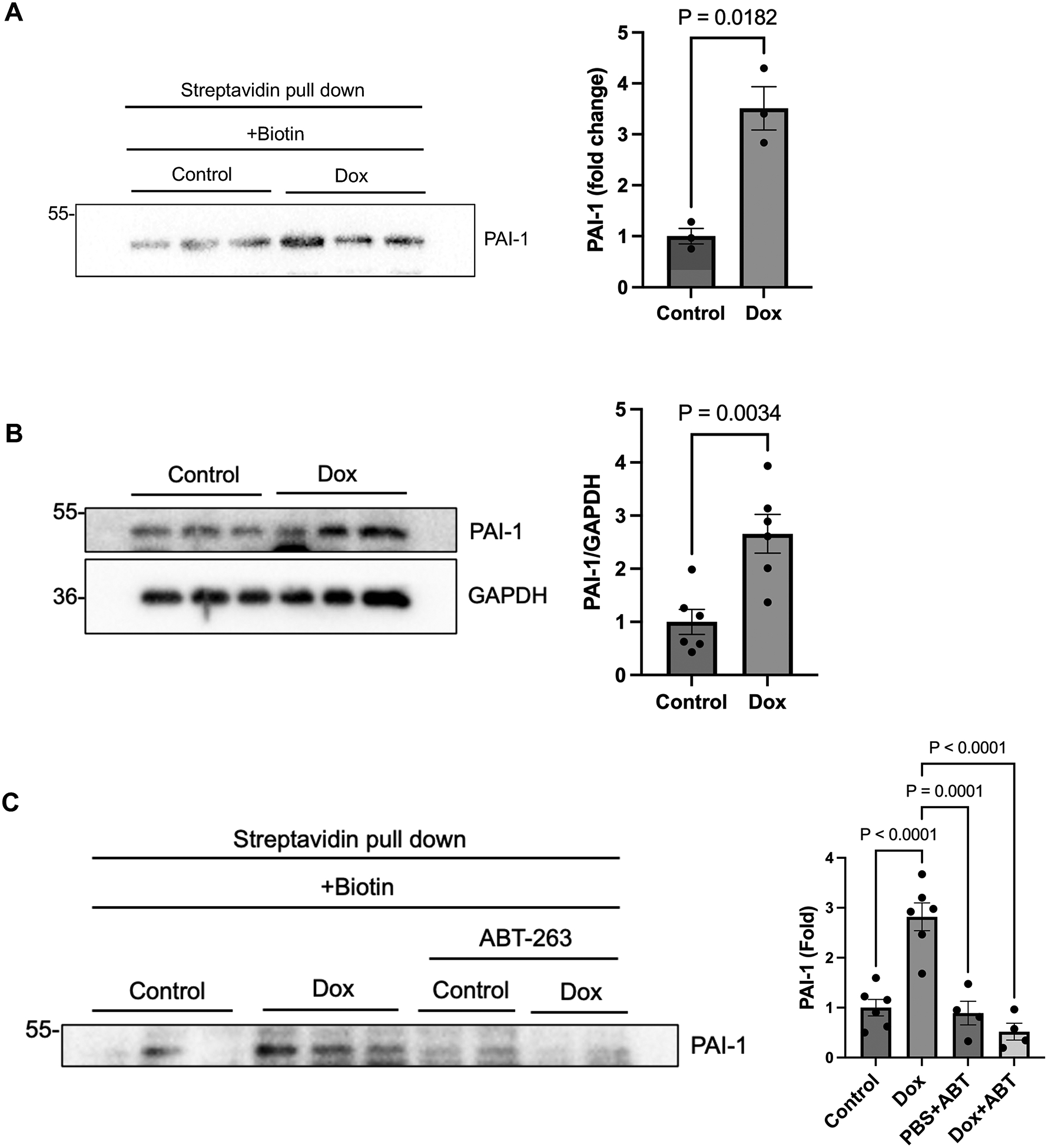
Identification of PAI-1 as a secreted protein induced by Dox using *cER-BioID* mice. (A) *Left:* Western blot analysis of PAI-1 in serum from *cER-BioID* mice. *Right:* Quantification of PAI-1 band intensity; (B) *Left:* Western blot analysis of PAI-1 in heart tissue lysates from control and Dox-treated mice. *Right:* Quantification of PAI-1 band intensity. Mice were treated with PBS + biotin (control) or Dox + biotin; (C) *Left:* Western blot analysis of PAI-1 in serum from *cER-BioID* mice. *Right:* Quantification of PAI-1 band intensity. Mice were treated with PBS + biotin (control), Dox + biotin, PBS + ABT-263 + biotin, or Dox + ABT-263 + biotin. *n* = 3 per group in (A); *n* = 6 per group in (B); *n* = 6 for PBS + biotin and Dox + biotin groups in (C); *n* = 4 for PBS + ABT-263 + biotin and Dox + ABT-263 + biotin groups in (C). Data are presented as mean ± SEM. Statistical significance was assessed using unpaired Student’s *t*-test or one-way ANOVA followed by Bonferroni–Dunn *post hoc* test. *P* < 0.05 was considered statistically significant. Dox: doxorubicin; *cER-BioID*: cardiac-specific endoplasmic reticulum-BioID; PAI-1: plasminogen activator inhibitor-1; PBS: phosphate-buffered saline; *n:* the number of mice; SEM: standard error of the mean; ANOVA: analysis of variance.

We confirmed that Dox- and biotin-treated *cER-BioID* mice exhibit significantly elevated serum PAI-1 levels compared to PBS- or PBS + biotin-treated controls by purifying biotinylated proteins and performing immunoblot analyses with an anti-PAI-1 antibody (Figure 1A and Figure S1E). Similarly, cardiac tissues from Dox-treated mice exhibited elevated PAI-1 protein expression compared to those from untreated mice (Figure 1B). To assess whether PAI-1 production occurs in senescent cells, we co-administered ABT-263 (50 mg/kg orally for five days), a senolytic compound^[21]^, along with Dox (Figure S1F). We have shown previously that ABT-263 inhibits Dox-induced senescence of cardiomyocytes and cardiomyopathy in mice^[14]^. The serum PAI-1 level of Dox-, ABT-263-, and biotin-treated *cER-BioID* mice was significantly lower than that of Dox- and biotin-treated mice without ABT-263 treatment (Figure 1C). The results suggest that Dox-induced production and secretion of PAI-1 occurs in senescent cardiomyocytes.

### 3.2 PAI-1 secreted from senescent cardiomyocytes spreads senescence to neighboring cells

**Figure 2.**
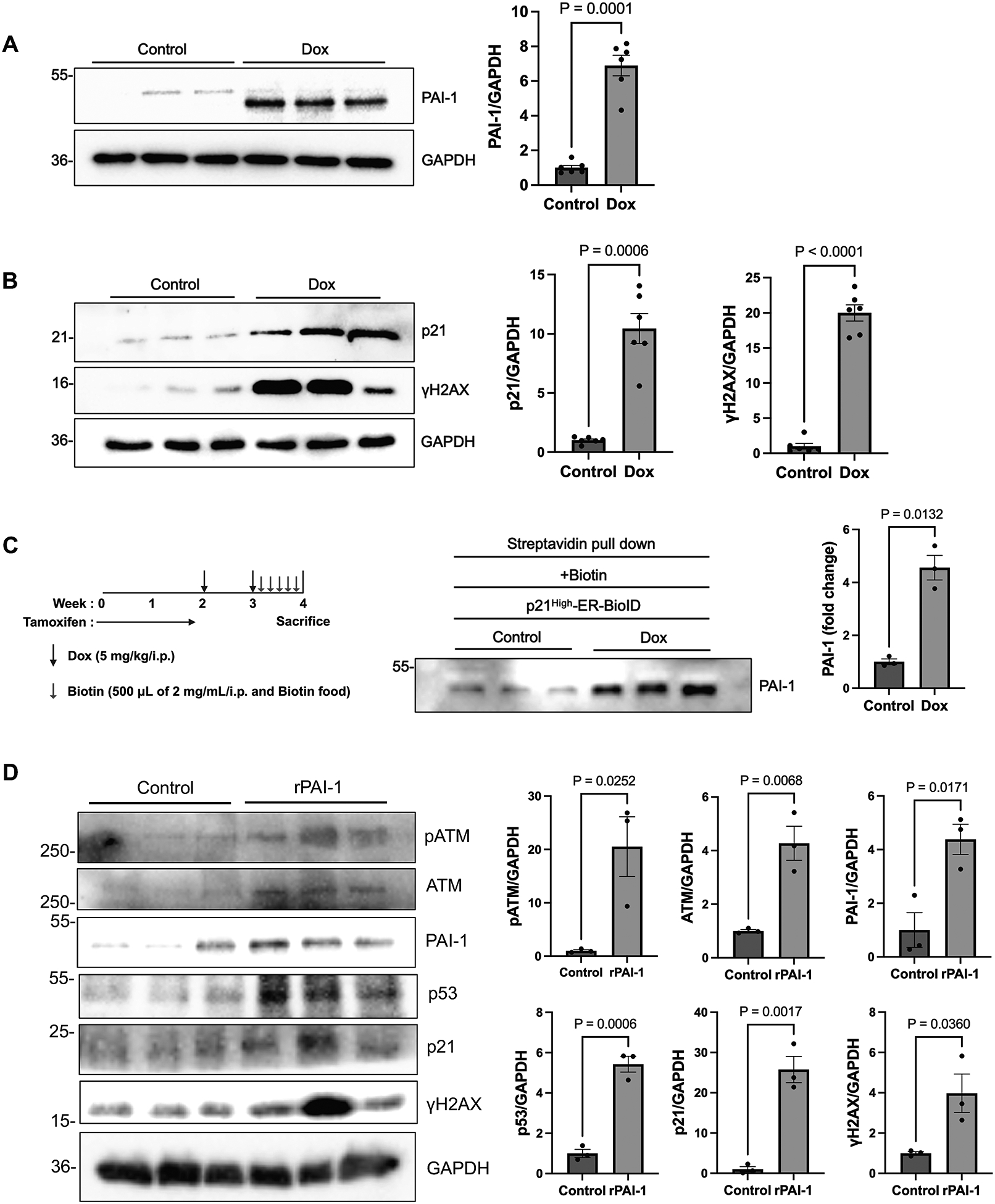
PAI-1 and other cellular senescence markers are elevated in cardiomyocytes exposed to Dox. (A) *Left:* Western blot analysis of PAI-1 in NRVMs treated with PBS (control) or Dox. *Right:* Quantification of PAI-1 band intensity; (B) *Left:* Western blot analysis of p21 and γH2AX in control or Dox-treated NRVMs. *Right:* Quantification of p21 and γH2AX band intensity; (C) *Left:* Schematic representation of the tamoxifen, Dox, and biotin administration protocol in *p21*^*High*^-*ER-BioID* mice. *Center:* Western blot analysis of PAI-1 in serum from *p21*^*High*^-*ER-BioID* mice treated with PBS + biotin (control) or Dox + biotin. *Right:* Quantification of PAI-1 band intensity; (D) NRVMs were treated with 10 ng of recombinant human PAI-1 protein for 72 h. *Left:* Western blot analysis of pATM, ATM, PAI-1, p53, p21, and γH2AX in NRVMs treated with rPAI-1 or vehicle (control). *Right:* Quantification of band intensity for each protein. *n* = 6 per group in (A) and (B); *n* = 3 per group in (C) and (D). Data are presented as mean ± SEM. Statistical significance was assessed using unpaired Student’s *t*-test or one-way ANOVA followed by Bonferroni–Dunn *post hoc* test. *P* < 0.05 was considered statistically significant. NRVMs: neonatal rat ventricular cardiomyocytes; PBS: phosphate-buffered saline; Dox: doxorubicin; PAI-1: plasminogen activator inhibitor-1; *ER*: endoplasmic reticulum; rPAI-1: recombinant human PAI-1 protein; SEM: standard error of the mean; ANOVA: analysis of variance.

To determine whether Dox directly induces upregulation and secretion of PAI-1 in cardiomyocytes in a cell-autonomous manner, we treated NRVMs with Dox (100 nM for 72 hours) or PBS and evaluated the levels of senescence markers. Dox treatment significantly increased the expression of both PAI-1 (Figure 2A) and senescence markers, including p21 and γH2AX (Figure 2B), indicating that Dox upregulates both senescence and the production of PAI-1 in cardiomyocytes. To confirm that Dox upregulates PAI-1 in cardiomyocytes in the heart *in vivo*, we conducted single cell RNA sequencing analyses of mouse hearts with or without Dox treatment (Figure S4). mRNA expression of *Serpine1 (Pai1)* was significantly upregulated in the cardiomyocyte fraction in response to Dox treatment. Interestingly, upregulation of *p53* and downregulation of plasminogen activator 1 (*Plat*) were also observed in the cardiomyocyte fraction.

To further investigate whether PAI-1 is specifically secreted by senescent cells, we expressed the ER-BioID system under the control of the p21^*High*^ promoter, which is known to be activated in a senescent cell-specific manner^[12]^. To this end, we generated *p21*^*High*^-*ER-BioID* mice by crossing *stop-flox-ER BioID*^*HA*^ knock-in mice with *p21*^*High*^-*Cre-ERT2* mice. Following treatment with tamoxifen, Dox, and biotin, serum from Dox + biotin-treated *p21*^*High*^-*ER-BioID* mice showed significantly elevated biotinylated PAI-1 levels compared to controls (Figure 2C). These results indicate that Dox-induced PAI-1 secretion occurs from senescent cells. Based on these findings, we hypothesized that senescent cardiomyocytes may act in an autocrine/paracrine manner to induce senescence in the local tissue. To test this hypothesis, we treated NRVMs with recombinant human PAI-1 protein (rPAI-1). This treatment resulted in an increase in multiple senescence markers, including p53, p21, and γH2AX (Figure 2D), suggesting that extracellular PAI-1 induces senescence in surrounding cells. rPAI-1 also upregulated PAI-1 itself in NRVMs, suggesting the presence of the self-amplification mechanism commonly observed in SASP factors^[22]^. Interestingly, rPAI-1 also upregulated Ataxia-telangiectasis mutated (ATM) kinase and its phosphorylated form, suggesting that ATM is activated to facilitate DNA repair mechanisms^[23]^. Consistent with our hypothesis that Dox-induced senescence spreads via an autocrine/paracrine mechanism, the supernatant of NRVMs treated with Dox was able to upregulate PAI-1 and senescence markers when transferred to NRVMs cultured in different dishes. In contrast, the supernatant from NRVMs treated with Dox in the presence of ABT-263 failed to upregulate either PAI-1 or senescence markers (Figure S5).

### 3.3 Inhibition of PAI-1 attenuates cardiomyocyte senescence and enhances apoptosis signaling

**Figure 3.**
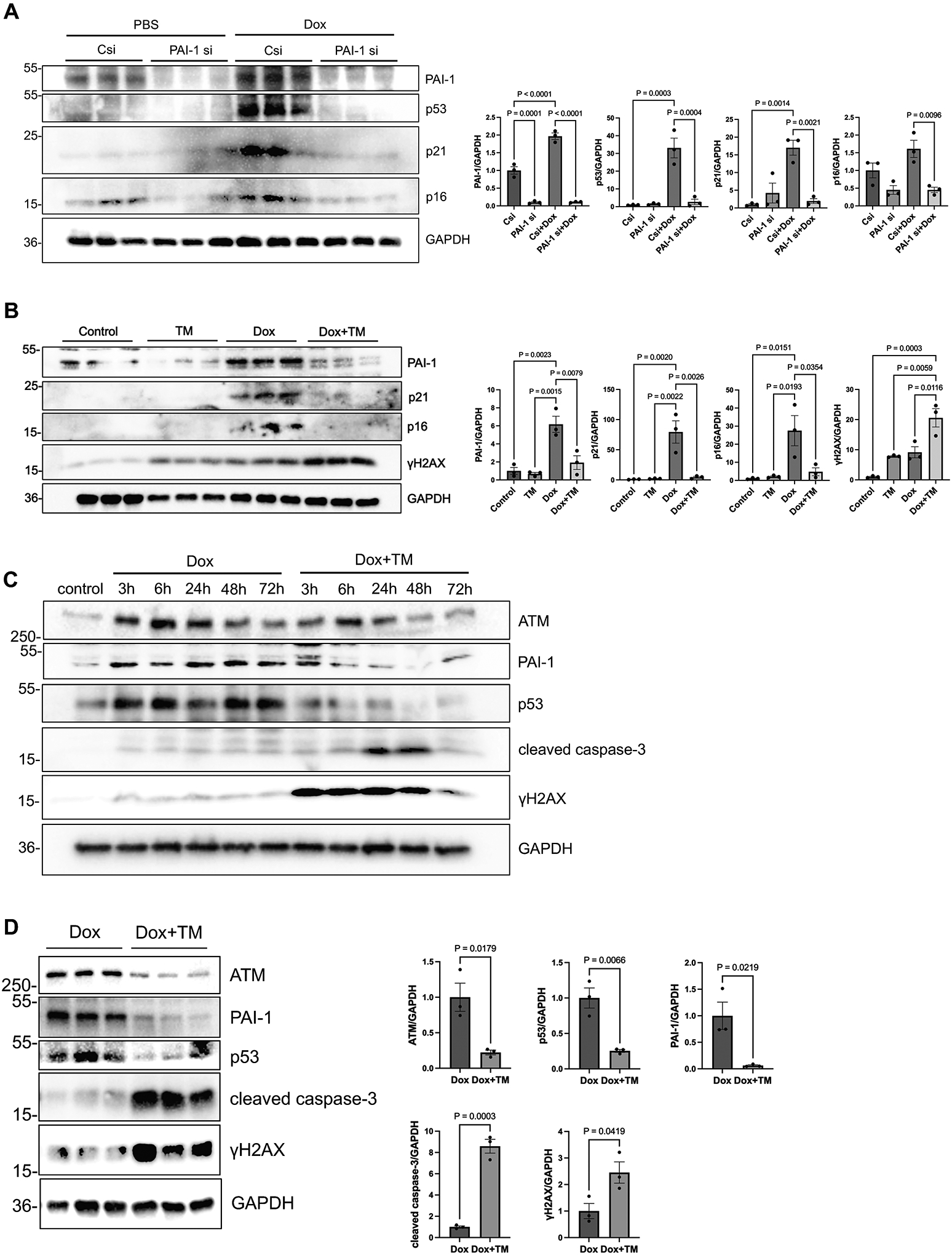
Doxorubicin-induced cardiomyocyte senescence involves PAI-1 and is attenuated by TM5275. (A) *Left:* Western blot analysis of PAI-1, p53, p21, and p16 in NRVMs treated with PBS (control), Csi, or PAI-1 si, with or without Dox (100 nM). *Right:* Quantification of band intensity for each protein; (B) NRVMs were treated with Dox (100 nM) and/or TM5275 (25 μM) for 72 hours. *Left:* Western blot analysis of PAI-1, p21, p16, and γH2AX in NRVMs treated with vehicle (control), TM5275, Dox, or Dox + TM5275. *Right:* Quantification of band intensity for each protein; (C) Western blot analysis of time-dependent expression of ATM, p53, cleaved caspase-3, and γH2AX in NRVMs treated with Dox (100 nM) or Dox (100 nM) + TM5275 (25 μM); (D) *Left:* Western blot analysis of ATM, p53, cleaved caspase-3, and γH2AX in NRVMs treated with Dox (100 nM) or Dox (100 nM) + TM5275 (25 μM) for 24 hours. *Right:* Quantification of band intensity for each protein. *n* = 4 per group in (A); *n* = 3 per group in (B); *n* = 1 per group in (C); *n* = 3 per group in (D). Data are presented as mean ± SEM. Statistical significance was assessed using unpaired Student’s *t*-test or one-way ANOVA followed by Bonferroni–Dunn *post hoc* test. *P* < 0.05 was considered statistically significant. PAI-1: plasminogen activator inhibitor-1; NRVMs: neonatal rat ventricular myocytes; Csi: control siRNA; PAI-1 si: PAI-1 siRNA; Dox: doxorubicin; ATM: Ataxia-telangiectasis mutated; SEM: standard error of the mean; ANOVA: analysis of variance.

To examine whether PAI-1 plays a functional role in mediating senescence in cardiomyocytes, we performed gene silencing in NRVMs using siRNA targeting PAI-1 (*Pai-1*si). After 48 hours, cardiomyocytes were treated with Dox for 72 hours to induce senescence. Treatment with *Pai-1*si downregulated PAI-1 protein levels and key senescence markers, including p53, p21, and p16, in the presence of Dox (Figure 3A), indicating that endogenous PAI-1 contributes to either the establishment or the maintenance of senescence in cardiomyocytes during Dox treatment.

We next assessed the effect of pharmacological inhibition of PAI-1 on Dox-induced senescence. NRVMs were treated with Dox in the presence or absence of TM5275, a selective PAI-1 inhibitor^[24]^. Although Dox treatment for 72 hours increased the protein levels of PAI-1, p21, and p16, co-treatment with TM5275 suppressed the upregulation of both PAI-1 and the senescence markers, with the exception of γH2AX (Figure 3B).

To directly assess cellular senescence, we performed SA-β-gal staining in NRVMs. Dox treatment significantly increased the proportion of SA-β-gal-positive cells, whereas PAI-1 inhibition with TM5275 markedly reduced the SA-β-gal positivity (Figure S6A,B), confirming attenuation of Dox-induced senescence. Importantly, to determine whether PAI-1 inhibition interferes with the antitumor efficacy of Dox, we assessed Dox-induced cytotoxicity in EO771 breast cancer cells. TM5275 did not affect Dox-induced cytotoxicity in EO771 cells (Figure S6C), indicating that PAI-1 inhibition does not compromise the anticancer effects of Dox.

We further evaluated the time-dependent effects of PAI-1 inhibition in NRVMs. Dox treatment rapidly upregulated p53 within 3 hours, whereas TM5275 inhibited Dox-induced upregulation of p53. Interestingly, while Dox increased the expression of γH2AX, a marker of DNA double-strand breaks, TM5275 enhanced its elevation, starting as early as after 3 hours of Dox treatment, with attenuation observed at 72 hours. This suggests that DNA damage is promoted in the presence of PAI-1 inhibition. In parallel, cleaved caspase-3 levels were increased transiently between 24 and 48 hours post-treatment with Dox + TM5275 (Figure 3C), indicating that PAI-1 inhibition promotes apoptosis. Additional Western blot analyses using samples collected 24 hours after treatment were consistent with the initial findings, showing significant downregulation of ATM and p53 levels and significant upregulation of cleaved caspase-3 and γH2AX levels in the Dox + TM5275 group compared with the Dox group (Figure 3D). The promotion of DNA damage and apoptosis in the presence of PAI-1 inhibition despite suppression of senescence markers is counterintuitive. However, senescent cells activate cell survival mechanisms despite DNA damage, and the DNA damage response can activate a series of events promoting DNA repair, including activation of ATM. In fact, rPAI-1 activated ATM (Figure 2D), whereas TM5275 downregulated ATM (Figure 3C,D). Thus, although Dox-induced secretion of PAI-1 promotes the accumulation of senescent cells by promoting their survival, PAI-1 inhibition modifies the cellular response to DNA damage, shifting it from a senescence-associated survival program toward increased apoptotic signaling and preventing stable senescence. This altered balance between senescence and apoptosis may contribute to the protective effects of PAI-1 inhibition in Dox-treated cardiomyocytes.

### 3.4 PAI-1 inhibition mitigates Dox-induced cardiac dysfunction and remodeling *in vivo*

**Figure 4.**
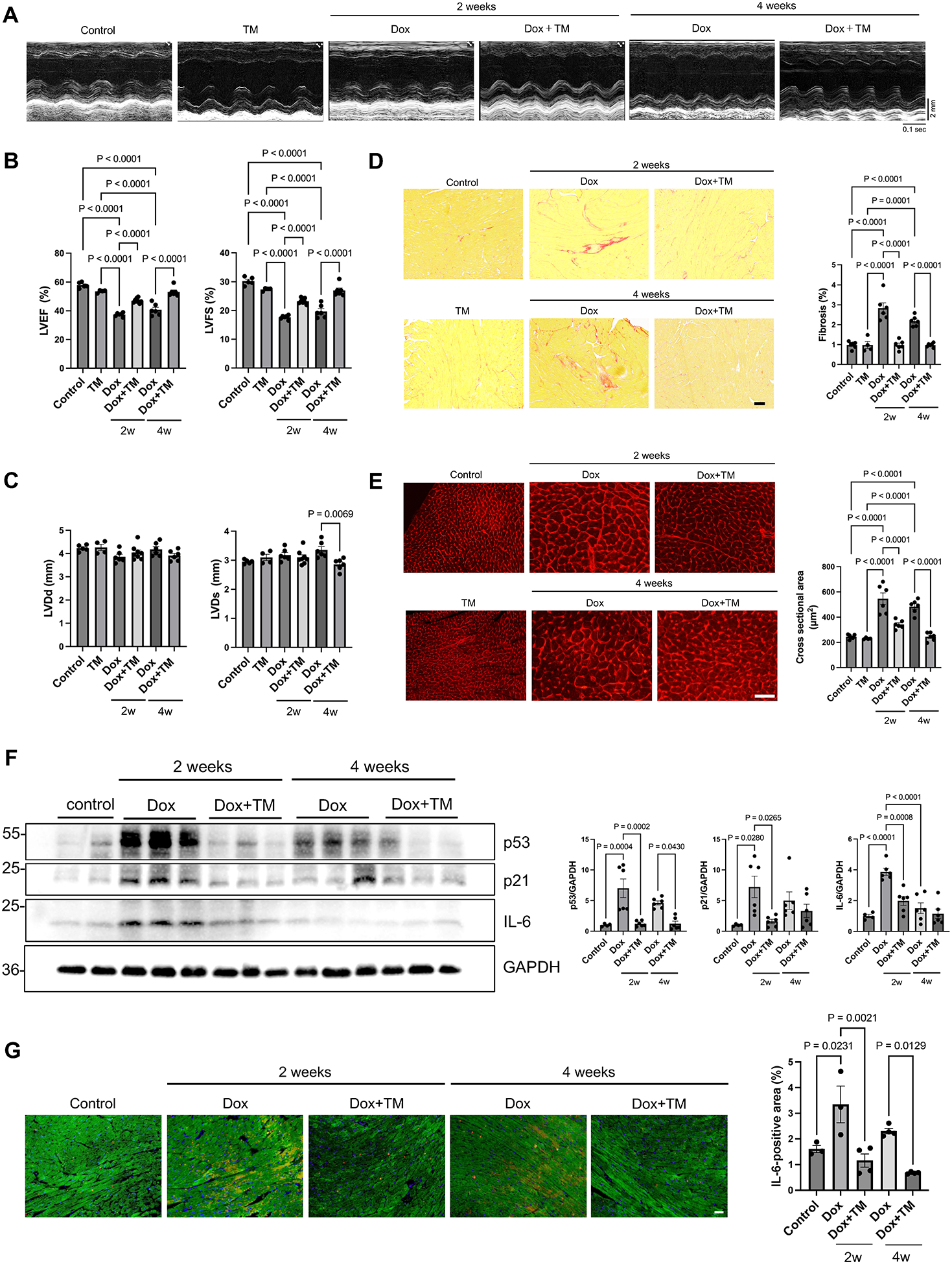
TM5275 improves cardiac function and reduces senescence- and stress-related markers in a mouse model of doxorubicin-induced cardiotoxicity. Wild-type mice were treated with Dox in the presence or absence of TM5275. Dox was injected two times, at weeks 0 and 1 (i.p. injection, 5 mg/kg), in either the presence or absence of TM5275 for 2 weeks (10 mg/kg orally). One group of mice was sacrificed at 2 weeks to evaluate the effect of Dox and TM5275 in the acute phase and another group of mice was sacrificed at 4 weeks to evaluate the chronic effect. (A) Representative M-mode echocardiographic images from control, TM5275, Dox, and Dox + TM5275 mice at 2 weeks and 4 weeks. Scale bar = 100 μm; (B) LVEF and LVFS in control, TM5275, Dox, and Dox + TM5275 groups at 2 weeks and 4 weeks; (C) LVDd and LVDs in control, Dox, and Dox + TM5275 groups at 2 weeks and 4 weeks; (D) *Left:* Representative images of PASR staining of cardiac tissue in control, TM5275, Dox, and Dox + TM5275 groups at 2 weeks and 4 weeks. Scale bar = 100 μm. *Right:* Quantification of cardiac fibrosis; (E) *Left:* Representative images of WGA staining in control, TM5275, Dox, and Dox + TM5275 groups at 2 weeks and 4 weeks. Scale bar: 50 μm. *Right:* Quantification of cardiac cross-sectional area; (F) *Left:* Western blot analysis of p53 in control, Dox, and Dox + TM5275 groups at 2 weeks and 4 weeks. *Right:* Quantification of band intensity; (G) *Left:* Representative images of IL-6 (red) and cTNT (green) in control, Dox, and Dox + TM5275 groups at 2 weeks and 4 weeks. Scale bar = 20 μm. *Right:* Percentage of IL-6 positive area. *n* = 5 for control group, *n* = 4 for TM5275 group, *n* = 6 for Dox, and Dox + TM5275V groups in (B) and (C); *n* = 6 for control, Dox, and Dox + TM5275 groups, *n* = 4 for TM5275 group in (D) and (E); *n* = 4 for control, *n* = 6 for Dox and Dox + TM5275 groups at 2 weeks and 4 weeks in (F); *n* = 4 for control and Dox at 2 weeks, and *n* = 4 for Dox + TM5275 at 2 weeks, Dox at 4 weeks, and Dox + TM5275 at 4 weeks in (G). Data are presented as mean ± SEM. Statistical significance was assessed using one-way ANOVA followed by Bonferroni–Dunn *post hoc* test. *P* < 0.05 was considered statistically significant. Dox: doxorubicin; LVEF: left ventricular ejection fraction; LVFS: left ventricular fractional shortening; LVDd: left ventricular end-diastolic diameter; LVDs: left ventricular end-systole; PASR: picric acid Sirius red; WGA: wheat germ agglutinin; SEM: standard error of the mean; ANOVA: analysis of variance.

To evaluate the cardioprotective effects of PAI-1 inhibition *in vivo*, wild-type mice were treated with Dox in the presence or absence of TM5275. Dox was injected twice, at weeks 0 and 1 (i.p. injection, 5 mg/kg), in the presence or absence of TM5275 for 2 weeks (10 mg/kg/day orally). One group of mice was sacrificed at 2 weeks to evaluate the effect of Dox and TM5275 in the acute phase, whereas a second group was sacrificed at 4 weeks to evaluate the chronic effect (Figure S7A,B,C). At both time points, Dox treatment significantly reduced LVEF and fractional shortening compared to vehicle control treatment, but co-administration of TM5275 significantly improved systolic function after Dox treatment (Figure 4A,B). Left ventricular systolic diameter was significantly increased in Dox-treated mice compared to in Dox + TM5275-treated mice (Figure 4C), consistent with the improvement of left ventricular function in the presence of TM5275. Although the heart weight-to-body weight ratio (HW/BW) did not differ significantly among the groups (Figure S7C), histological analyses revealed enhanced cardiac fibrosis and increased cardiomyocyte cross-sectional area in Dox-treated mice, both of which were attenuated by TM5275 under both acute and chronic conditions (Figure 4D,E). These findings demonstrate that endogenous PAI-1 contributes to Dox-induced cardiotoxicity, including systolic dysfunction, fibrosis, and cardiomyocyte hypertrophy. Dox-induced increases in p53 and IL-6 expression in the heart were also suppressed in the presence of TM5275, as evaluated with immunoblot analyses of p53 (Figure 4F) and double staining of myocardial sections with anti-IL-6 and anti-cTNT antibodies (Figure 4G). These results are consistent with the notion that PAI-1 inhibition reduces the level of senescence in the heart during Dox treatment.

### 3.5 PAI-1 inhibition may promote apoptosis of cardiomyocytes with persistent DNA damage while reducing overall cell death

**Figure 5.**
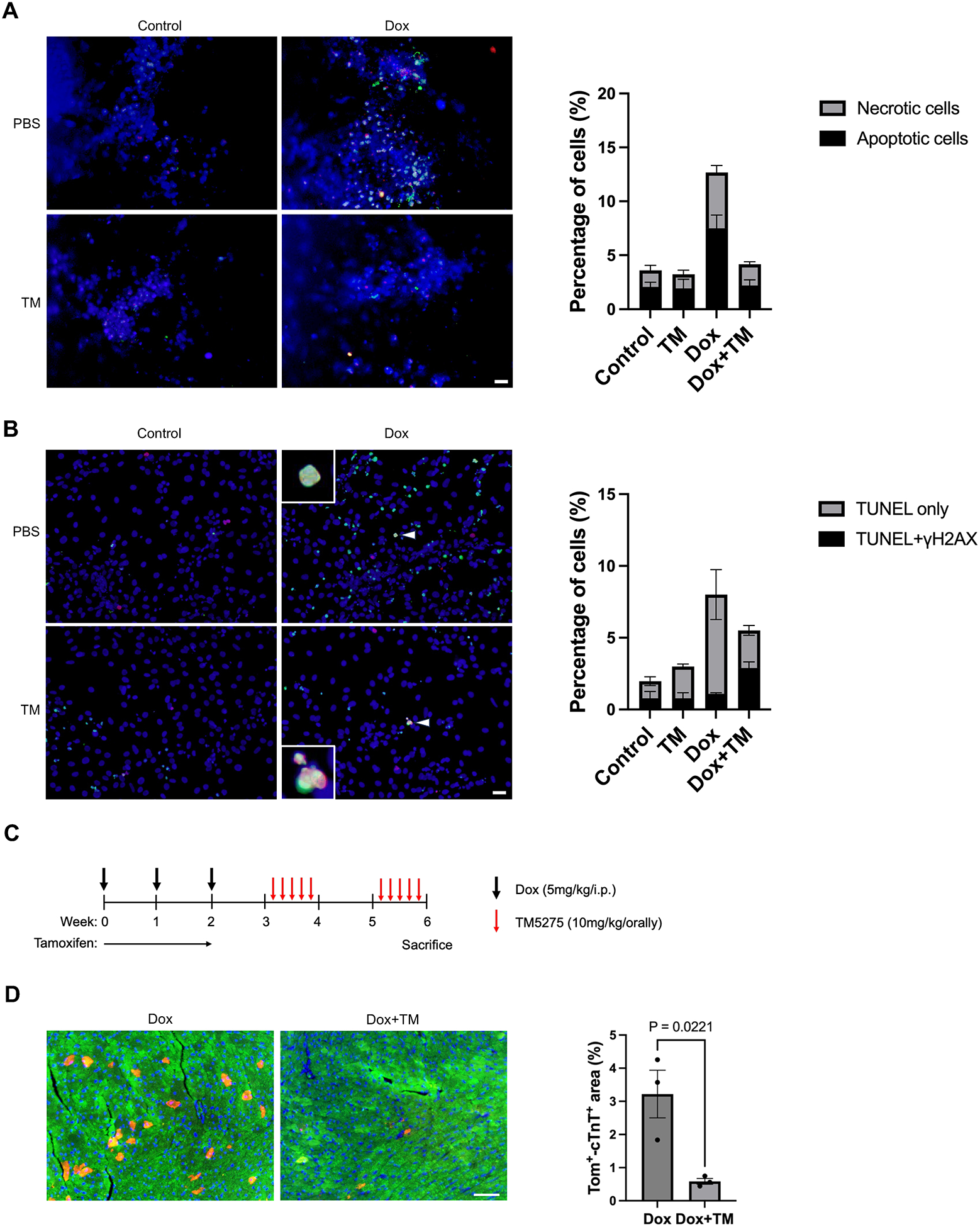
PAI-1 inhibition promotes apoptosis and clearance of senescent cardiomyocytes after exposure to doxorubicin. (A) NRVMs were treated with Dox (100 nM) and/or TM5275 (10 μM) for 3 hours. *Left:* Representative images of live/dead cell staining showing healthy cells (blue), apoptotic cells (green), and necrotic cells (red) in NRVMs treated with vehicle (control), TM5275, Dox, or Dox + TM5275. Scale bar = 100 μm. *Right:* Quantification of the proportions of healthy, apoptotic, and necrotic cells in each group; (B) NRVMs were treated with Dox (100 nM) and/or TM5275 (10 μM) for 3 hours. *Left:* Representative immunofluorescence images of TUNEL (green), γH2AX (red), and DAPI (blue) staining in NRVMs treated with vehicle (control), TM5275, Dox, and Dox + TM5275. *Right:* Quantification of TUNEL-positive only and double-positive (TUNEL and γH2AX) NRVMs in each treatment group. Scale bar = 100 μm. Arrow heads point to TUNEL and γH2AX double positive cells; (C) Schematic representation of the tamoxifen, Dox, and TM5275 administration protocol used in *p21^High^*-tdTomato mice; (D) *Left:* Representative immunofluorescence images of tdTomato (red), cTnT (green), and DAPI (blue) staining in heart sections from *p21^High^-tdTomato* mice. Scale bar = 50 μm. *Right:* Quantification of the area positive for both tdTomato and cTnT. Five random fields from 3 samples were analyzed per group. Data are presented as mean ± SEM. Statistical significance was assessed using one-way ANOVA followed by Bonferroni–Dunn *post hoc* test. *P* < 0.05 was considered statistically significant. NRVMs: neonatal rat ventricular myocytes; Dox: doxorubicin; TUNEL: TdT-mediated dUTP-biotin nick end labeling; SEM: standard error of the mean; ANOVA: analysis of variance.

We next examined whether PAI-1 inhibition modulates the fate of cardiomyocytes under Dox treatment. Using a Cell Meter^™^ apoptotic and necrotic multiplexing detection assay, we found that TM5275 did not affect apoptosis or necrosis in NRVMs in the absence of Dox. In Dox-treated cells, TM5275 reduced overall apoptosis and necrosis (Figure 5A), indicating attenuation of net cell death. To determine whether the inhibition of cell death is seen uniformly in every cell type, we performed γH2AX/TUNEL co-staining. Although TM5275 reduced the number of TUNEL-positive/γH2AX-negative cardiomyocytes, it increased the number of TUNEL- and γH2AX-double positive cardiomyocytes (Figure 5B). These findings suggest that PAI-1 inhibition preferentially promotes apoptosis in cardiomyocytes with persistent DNA damage, while it inhibits apoptosis in less-damaged cells. Collectively, PAI-1 inhibition shifts the fate of severely damaged cardiomyocytes toward apoptosis, thereby reducing the overall cell loss.

### 3.6 TM5275 selectively reduces *p21* ^*High*^ senescent cardiomyocytes *in vivo*

To determine whether TM5275 alters the balance between senescence and apoptosis in cardiomyocytes *in vivo*, we generated *p21*^*High*^-*tdTomato* mice by crossing *p21*^*High*^-*CreERT2-tdTomato* mice^[25]^ with *Rosa-CAG-LSL-tdTomato* mice. In this model, senescent cells expressing high levels of p21 are labeled with tdTomato following consumption of tamoxifen-containing chow. To assess the impact of TM5275 on senescent cardiomyocytes, *cp21-tdTomato* mice were fed tamoxifen chow and then treated with Dox (i.p. injection, 5 mg/kg), with or without co-administration of TM5275 (10 mg/kg orally) (Figure 5C). Histological analysis revealed that mice receiving both Dox and TM5275 exhibited a smaller tdTomato-positive and cTNT-positive cardiomyocyte area than those treated with Dox alone (Figure 5D). These findings indicate that TM5275 reduces the number of *p21*^*High*^ cardiomyocytes in the Dox-treated heart *in vivo*.

## 4. Discussion

Using a cardiomyocyte-specific secretome profiling approach with *cER-BioID* mice, we identified PAI-1 as a key protein secreted by senescent cardiomyocytes following Dox treatment. Functionally, our findings indicate that PAI-1 contributes to Dox-induced cardiotoxicity *in vivo* by promoting and sustaining cellular senescence in the heart. Mechanistically, our data suggest that PAI-1 promotes senescence development and supports the survival of senescent cardiomyocytes.

Cellular senescence is characterized by the presence of the SASP, which involves the release of pro-inflammatory and tissue-remodeling factors^[26]^. The SASP profile is cell-type- and stimulus-specific, and the factors secreted by senescent cardiomyocytes during Dox-induced cardiomyopathy remain poorly characterized. Using the *cER-BioID* system, which labels proteins processed in the ER of cardiomyocytes, we cataloged proteins secreted via conventional pathways in response to Dox. Notably, PAI-1 (Serpin E1) and Alpha-1-antitrypsin 1–5 (Serpin A1e), both serpin family members, showed the highest upregulation, suggesting a shared role in Dox-induced cardiomyopathy. Driven by a *p21*^*High*^ promoter, a senescent cell-specific ER-BioID system also detected PAI-1 secretion, and inhibition of PAI-1 secretion with the senolytic agent ABT-263 confirmed that PAI-1 is secreted by senescent cardiomyocytes *in vivo*. Since PAI-1 induces senescence, the fact that PAI-1 is produced in senescent cells suggests that PAI-1 and senescence may stimulate one another.

At the functional level, siRNA-mediated knockdown of PAI-1 *in vitro* attenuated Dox-induced upregulation of senescence markers in cardiomyocytes. Consistent with these findings, pharmacological inhibition of PAI-1 with TM5275 *in vivo* attenuated Dox-induced upregulation of senescence markers in the heart (e.g., p53 and IL-6) and mitigated Dox-induced cardiomyopathy. These results support the notion that PAI-1 plays a functional role in mediating Dox-induced senescence in cardiomyocytes and consequent pathological cardiac remodeling and dysfunction. Importantly, under the conditions we tested, TM5275 did not attenuate Dox-induced cytotoxicity in EO771 murine breast cancer cells, indicating that PAI-1 inhibition alleviates Dox-induced cardiomyopathy without compromising antitumor efficacy.

Although PAI-1 has previously been shown to be involved in Dox-induced senescence in several cell types, including cardiomyocytes^[9,10]^, our results indicate that PAI-1 may also be involved in the increased survival of senescent cells, an important feature of senescence. Inhibition of PAI-1 by TM5275 treatment increased apoptosis in γH2AX-positive cardiomyocytes, suggesting that endogenous PAI-1 promotes resistance to apoptosis in cells experiencing persistent DNA damage. Accordingly, PAI-1 inhibition may reduce senescence in the Dox-treated heart by shifting the balance between survival and death toward apoptotic signaling preferentially in cardiomyocytes with persistent DNA damage, most likely in senescent cardiomyocytes, rather than broadly increasing cardiomyocyte death. Fate mapping experiments showed that the area of genetically tagged senescent cells was decreased, and other markers of senescence were downregulated at later time points in Dox-treated hearts in the presence of TM5275. Since senescence is a self-amplifying process, limiting the survival of senescent cells would be expected to slow the progression of senescence-associated pathology. Given the limited regenerative capacity of the adult heart^[27]^, a strategy that preferentially reduces senescent cardiomyocytes, rather than broadly increasing cardiomyocyte death, appears advantageous.

What is the mechanism through which PAI-1 inhibition promotes apoptotic signaling in senescent cells? A previous study showed that PAI-1 activates cell survival signaling pathways, including Akt and ERK1/2, and inhibits caspase-3^[28]^. In the present study, we show that recombinant PAI-1 activates ATM, a master regulator of DNA repair, in cultured NRVMs, suggesting that PAI-1 is sufficient to activate DNA repair signaling. Conversely, pharmacological inhibition of PAI-1 promoted γH2AX in the presence of Dox, accompanied by downregulation of ATM. A previous study showed that Serpine2 directly interacts with ATM, thereby stimulating phosphorylation and activation of ATM^[29]^. Since ATM allows senescent cells to repair damage and avoid apoptosis^[30,31]^, PAI-1 inhibition may promote apoptosis of senescent cells by inhibiting ATM.

It has been shown that p53 plays an important role in mediating transcription of *Pai-1* in fibroblasts in response to a carcinogen^[32]^. We found that Dox-induced upregulation of *Pai-1* in cardiomyocytes is accompanied by upregulation of *p53*. Furthermore, recombinant PAI-1 also upregulates p53. Thus, it is possible that p53 and PAI-1 stimulate one another. Together with the fact that inhibition of PAI-1 attenuates expression of PAI-1 in cardiomyocytes, this suggests that Dox-induced upregulation of PAI-1 initiates a self-amplification mechanism to promote PAI-1 and senescence through p53.

Several limitations of this study should be acknowledged. While *cER-BioID* enables cardiomyocyte-specific secretome profiling, it does not exclude contributions of PAI-1 secreted from other cardiac cell types or from extracardiac sources to Dox-induced cardiomyopathy. Because PAI-1 is produced by multiple cell populations, the cardioprotective effect of TM5275 may not be solely attributable to the inhibition of cardiomyocyte-derived PAI-1. Moreover, our analyses focused on early-phase injury and the use of a single model of cardiotoxicity. Long-term consequences of PAI-1 inhibition, particularly regarding cardiac regeneration and systemic effects, remain unclear. Additionally, while TM5275 is a selective inhibitor, off-target effects cannot be entirely ruled out. Future studies should explore the timing and duration of PAI-1 inhibition, and combination of PAI-1 inhibition and other cardioprotective or senescence-modulating strategies. Investigating the role of PAI-1 in other forms of cardiac stress or in human cardiomyocytes will also be crucial for clinical translation.

## 5. Conclusion

In summary, our study provides the proof-of-concept for utilizing *ER-BioID* mice in conjunction with other tools for investigating cellular senescence to identify cell-type-specific SASP factors in an unbiased manner. Our findings position PAI-1 as a critical mediator of Dox-induced cardiac dysfunction *in vivo*. PAI-1 promotes senescence in cardiomyocytes and supports the persistence of senescent cardiomyocytes by favoring their survival over apoptotic signaling, thereby contributing to maladaptive cardiac remodeling and functional decline. Inhibition of PAI-1 attenuates cellular senescence, enhances apoptotic signaling in cardiomyocytes with persistent DNA damage, and preserves cardiac structure and function without compromising the antitumor efficacy of Dox. These results provide a strong rationale for developing PAI-1-targeted therapies to mitigate chemotherapy-associated cardiac dysfunction by modulating the survival of senescent cardiomyocytes in Dox-induced cardiomyopathy hearts.

## Supplementary Material

Supplementary materials

The supplementary material for this article is available at: Supplementary materials.

## Data Availability

The data that support the findings, including statistical analyses, and reagents used are available from the corresponding author upon request.
