# Supplementary material for: Inhibition of PAI-1 shifts cardiomyocyte fate from senescence toward apoptosis and mitigates doxorubicin-induced cardiotoxicity
Source: Geromedicine. Author manuscript; Available in PMC 2026 Apr 29. (PMC13123424; doi:10.70401/geromedicine.2026.0018)
Supplement: Supplementary materials [file NIHMS2158405-supplement-Supplementary_materials.pdf]

Geromedicine
https://doi.org/10.70401/Geromedicine.2026.0018

**Supplementary information**

# Inhibition of PAI-1 shifts cardiomyocyte fate from senescence toward apoptosis and mitigates doxorubicin-induced cardiotoxicity

Yuka Shiheido-Watanabe, Eun-Ah Sung, Andreas Ivessa, Peiyong Zhai, Takuma Takada, Soichiro Ikeda, Masato Matsushita, Daniela Zablocki, Junichi Sadoshima

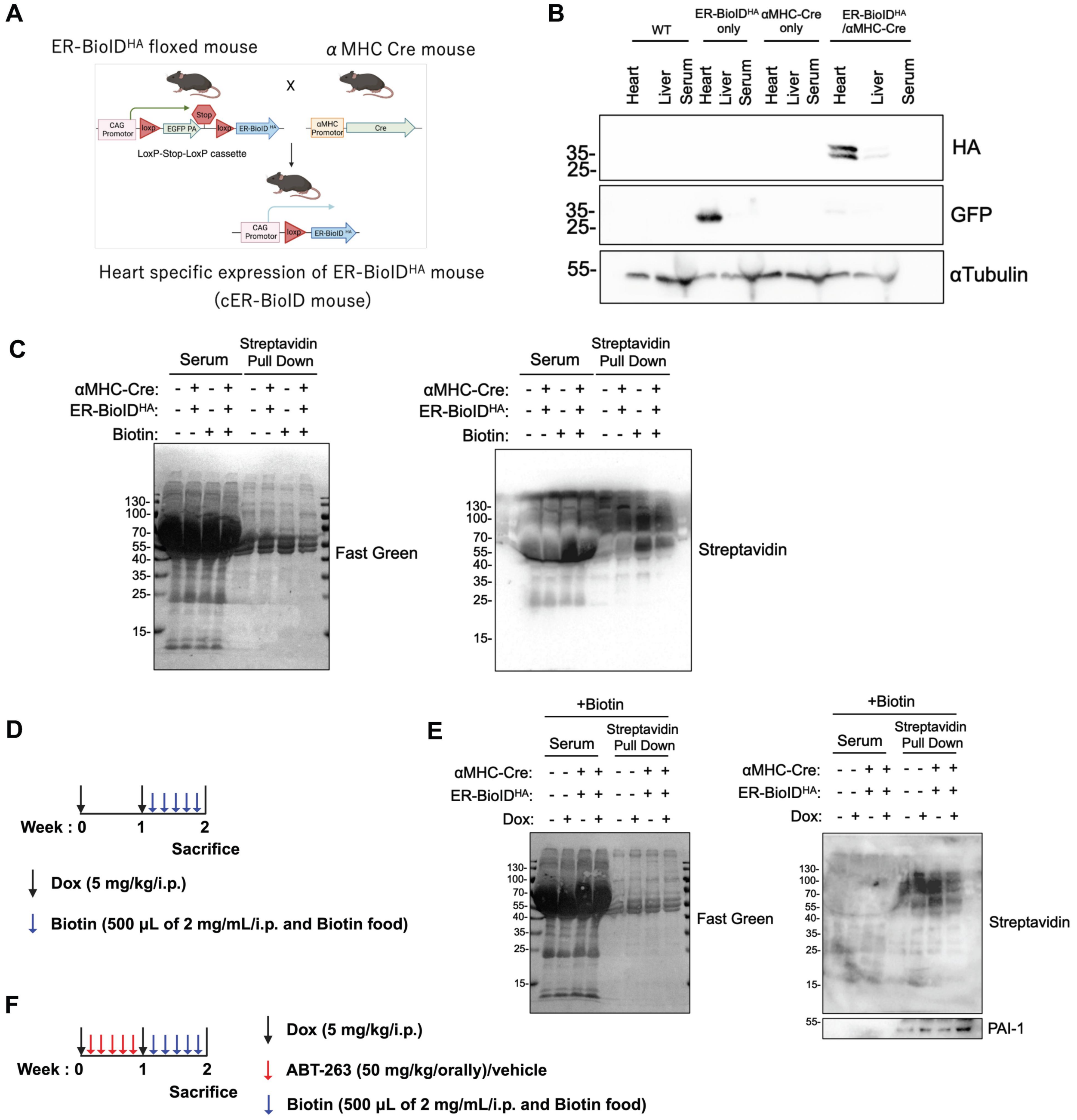


**Figure S1.** Characterization of the cardiac.specific secretome mouse and experimental protocols. (A) Generation of the cardiac-specific secretome mouse; (B) Cardiac-specific expression of cER-BioID was confirmed in αMHC-Cre-positive mice. Cre-mediated excision within tissues results in an inverse correlation between HA and GFP expression. αTubulin is shown as a loading control; (C) Detection of biotinylation in the srum of mice following biotin administration; (D) Schematic representation of the Dox and biotin administration protocol; (E) Dox-treated Myh6-Cre; ER-BioID mice exhibited increased PAI-1 level; (F) Experimental design for the combined administration of Dox, ABT-263, and biotin. Dox: doxorubicin.

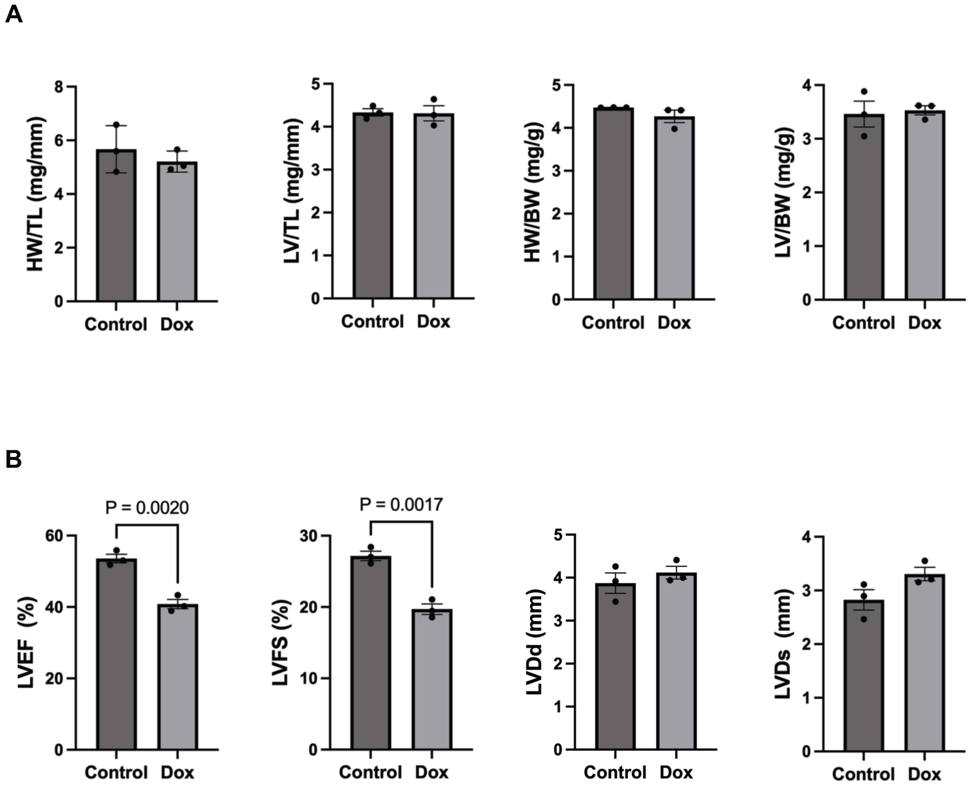


**Figure S2.** Validation of Dox.induced cardiac dysfunction in *cER.BioID* mice. (A) Body and organ weight indices in control and Dox-treated *cER-BioID* mice. HW heart weight; TL tibial length; LV left ventricular weight; BW body weight; (B) Echocardiographic assessment of cardiac function in control and Dox-treated *cER-BioID* mice. LVEF, LVFS, LVDd, and LVDs in control and Dox-treated *cER-BioID* mice. *n*= 3 per group. Data are presented as mean ± SEM. Statistical significance was assessed using unpaired Student's t-test. $P < 0.05$ was considered statistically significant. LVEF: left ventricular ejection fraction; LVFS: left ventricular fractional shortening; LVDd: left ventricular end-diastolic diameter; LVDs: left ventricular end-systole.

A

| PG.Protein Descriptions | PG.Genes | No. of Peptides | Ratio (PBS+Biotin/ PBS) | Ratio (Dox+Biotin/ PBS) | Ratio (Dox+Biotin/ PBS+Biotin) |
|---|---|---|---|---|---|
| Plasminogen activator inhibitor 1 | Serpine1 | 17 | 0.3 | 7.8 | 24.8 |
| Alpha-1-antitrypsin 1-5 | Serpina1e | 6 | 2.1 | 46.5 | 22.4 |
| Myomesin 2 | Myom2 | 58 | 0.1 | 2.6 | 20.6 |
| Cysteine-rich secretory protein 1 | Crisp1 | 2 | 1.6 | 14.9 | 9.0 |
| von Willebrand factor C and EGF domain-containing protein | Vwce | 1 | 0.3 | 1.9 | 5.7 |
| Ig kappa chain V-III region CBPC 101 | | 1 | 1.2 | 5.8 | 5.0 |
| Leucine-rich HEV glycoprotein | Lrg1 | 3 | 0.6 | 2.3 | 4.2 |
| Alpha-amylase 1 | Amy1;Amy2a5 | 2 | 1.1 | 4.2 | 3.9 |
| Heat shock 70 kDa protein 4 | Hspa4 | 3 | 0.6 | 2.0 | 3.4 |
| Polypyrimidine tract binding protein 3 | Ptbp3 | 1 | 1.2 | 3.7 | 3.0 |
| Haptoglobin | Hp | 2 | 1.6 | 4.3 | 2.7 |
| non-specific serine/threonine protein kinase | Obscn | 2 | 0.8 | 1.6 | 2.0 |
| Murinoglobulin-1 | Mug1 | 82 | 1.9 | 3.7 | 2.0 |
| cAMP-dependent protein kinase type II-beta regulatory subunit | Prkar2b | 7 | 0.9 | 1.6 | 1.8 |

B

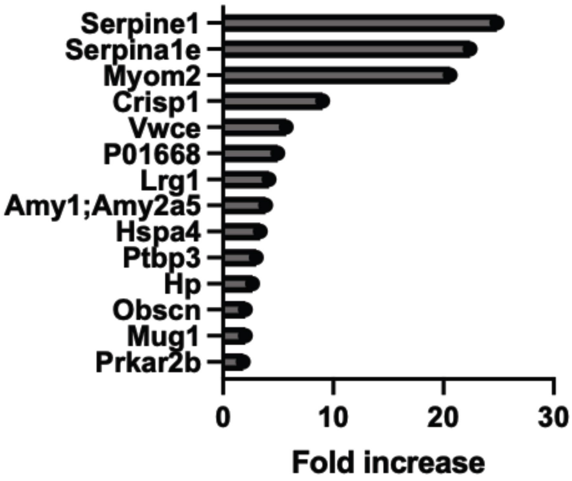


C

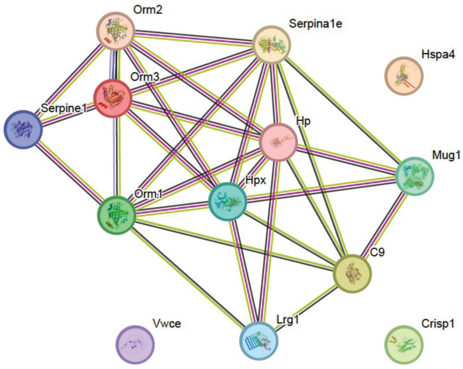


**Figure S3.** Identification of PAI-1 as a secreted protein induced by Dox using *cER.BioID* mice. (A) Mass spectrometry analysis of serum collected from mice treated with PBS, PBS + biotin, or Dox + biotin; (B) Secreted proteins ranked by fold change between Dox + biotin and PBS + biotin treatment groups; (C)Protein-protein interaction network of the identified secreted proteins generated by STRING analysis. Dox: doxorubicin; STRING: Search Tool for the Retrieval of Interacting Gene/Protein.

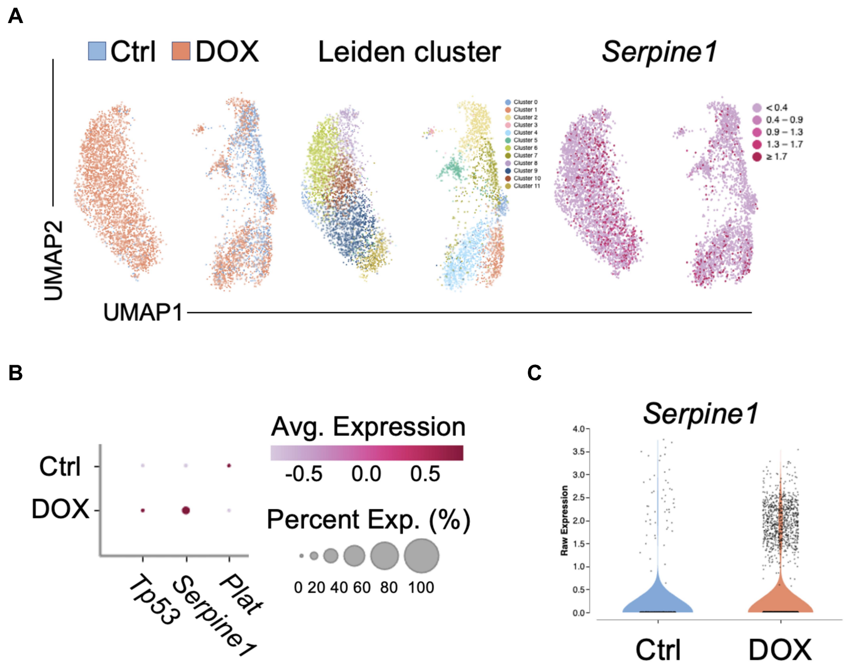


**Figure S4.** Dox treatment upregulated *Serpine1* expression in cardiomyocytes. (A) UMAP plots of single-cell transcriptomes from Ctrl (blue) and Dox-treated (orange) mouse hearts. Cells are annotated by treatment condition, Leiden clusters and *Serpine1* expression levels; (B) Dot plot showing expression of *Tp53* and *Serpine1* and *Plat* across conditions, where dot size represent the percentage of cells expressing each gene and color scale reflects average expression; (C) Violin plots comparing *Serpine1* expression in control versus Dox-treated cardiomyocytes. Ctrl: control; Dox: doxorubicin.

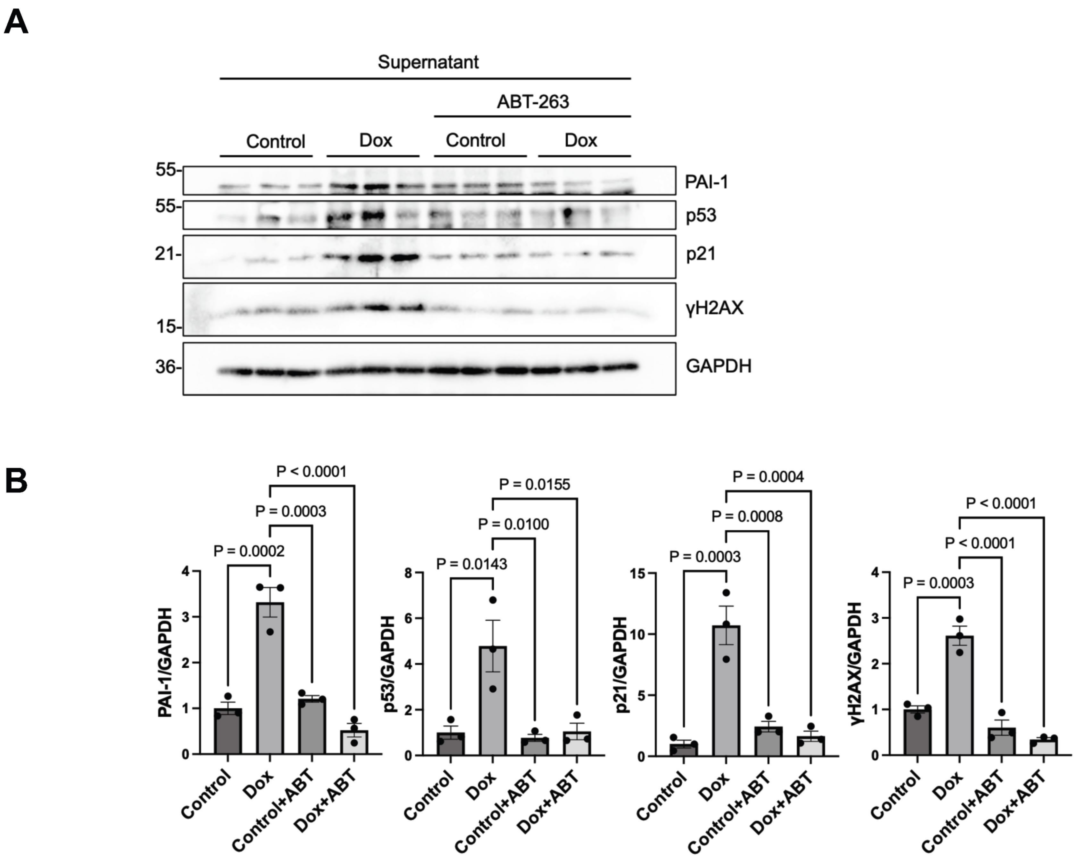


**Figure S5.** Supernatant from NRVMs treated with Dox in the presence of ABT.263 failed to upregulate senescence markers. (A) Western blot analysis of PAI-1, p53, p21, and γ H2AX in NRVMs treated with conditioned media collected from NRVMs exposed to PBS (Control), Dox, PBS + ABT-263, or Dox + ABT-263 for 72 hours; (B) Quantification of band intensity for each protein. *n* = 3 per group. Data are presented as mean ± SEM. Statistical significance was assessed using one-way ANOVA followed by Bonferroni–Dunn *post hoc* test. $P < 0.05$ was considered statistically significant.

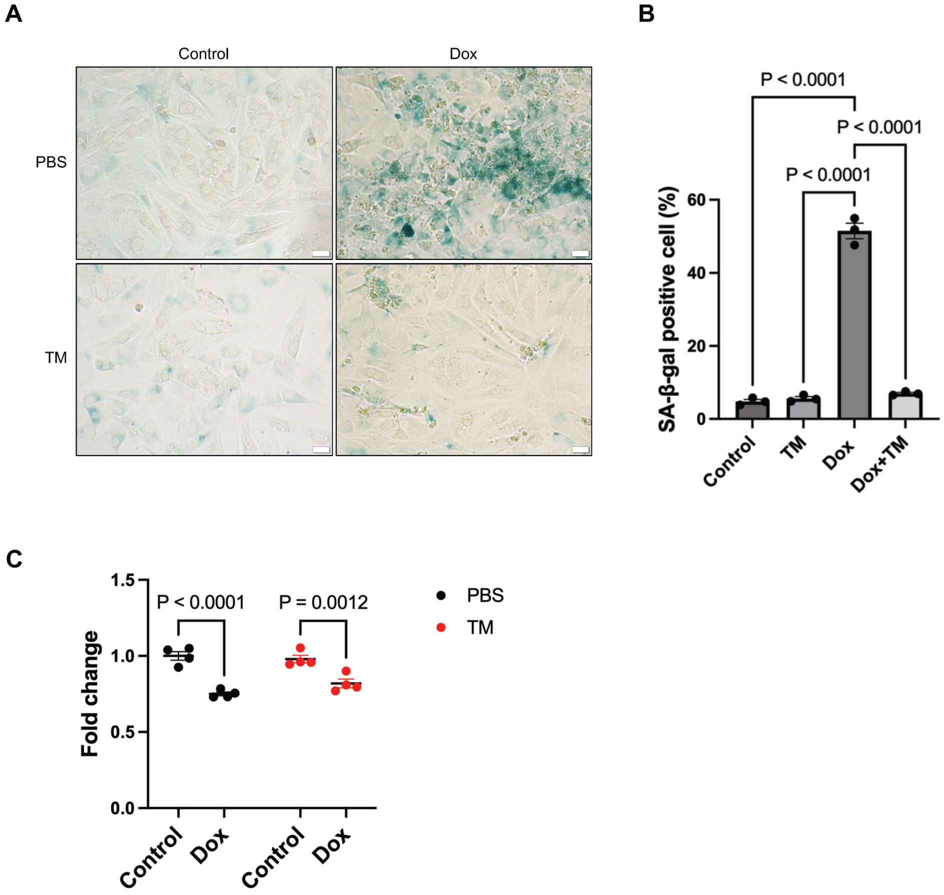


**Figure S6.** PAI-1 inhibition reduces cardiomyocyte senescence without affecting doxorubicin cytotoxicity in cancer cells. Neonatal rat ventricular myocytes (NRVMs) were treated with Dox (100 nM) and/or TM5275 (10uM) for 72 hours. (A) Representative images of SA-β-gal staining. Scale bar = 20 µm; (B) Quantification of SA-β-gal-positive cells; (C) EO771 murine breast cancer cells were treated with Dox (100 nM) in the presence or absence of the PAI-1 inhibitor TM5275 (25 µM) for 48 hours. Cell viability was assessed using CellTiter-blue assay. TM5275 did not attenuate Dox-induced cytotoxicity in EO771 cells. $n$ = 3 per group in (B); $n$=4 per group in (C). Data are presented as mean ± SEM. Statistical significance was assessed using one-way ANOVA followed by Bonferroni–Dunn *post hoc* test in (B); Statistical significance was assessed using two-way ANOVA followed by Sidak's multiple comparison test in (C). $P$ < 0.05 was considered statistically significant. Dox: doxorubicin; SA-β-gal: SA-β-galactosidase.

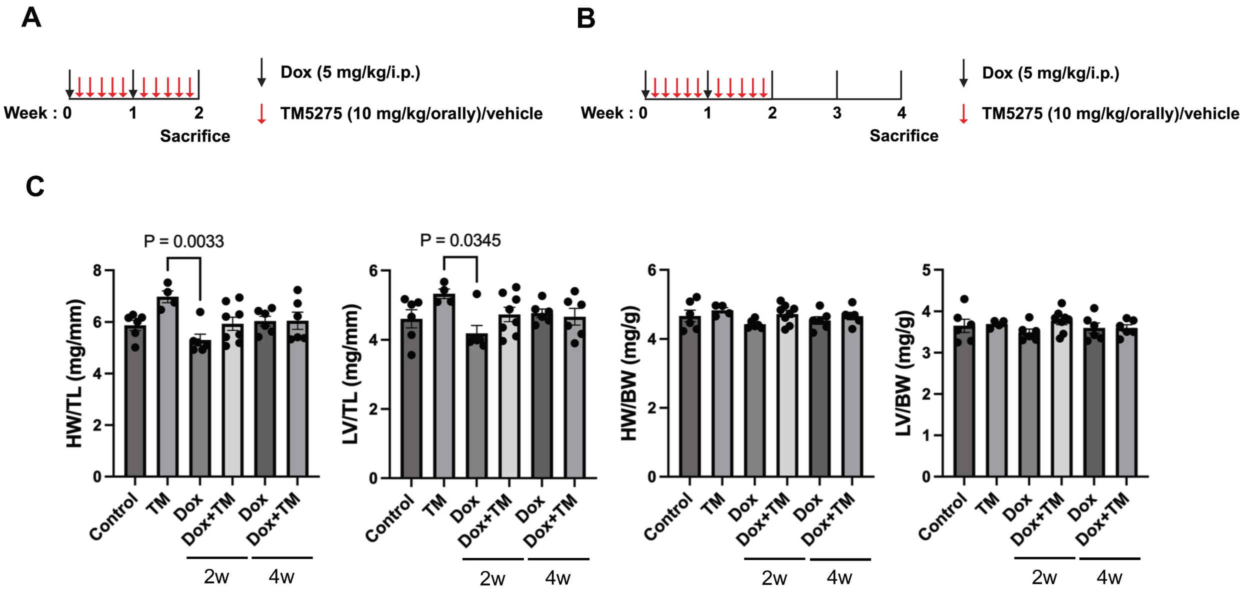


**Figure S7.** Experimental protocols and evaluation of cardioprotective effects of PAI-1 inhibition. (A) Schematic representation of the acute-phase protocol used to evaluate the cardioprotective effects of PAI-1 inhibition. Wild-type mice were treated with Dox with or without the PAI-1 inhibitor TM5275 during the acute phase, without a subsequent rest period; (B) Schematic representation of the delayed protocol. Mice received the same Dox and TM5275 treatments as in (A), followed by a 2-week rest period before evaluation; (C) Physiological measurements of control, TM5275-treated, Dox-treated, and Dox + TM5275-treated mice during the acute phase and after the 2-week rest period. HW heart weight; TL tibial length; LV left ventricular weight; BW body weight. $n = 6$ per group. Data are presented as mean ± SEM. Statistical significance was assessed using one-way ANOVA followed by Bonferroni–Dunn *post hoc* test. $P < 0.05$ was considered statistically significant. Dox: doxorubicin.
